# Hounsfield unit thresholds in differentiating adenoma from nonadenoma adrenal masses: a retrospective adrenalectomy-based study

**DOI:** 10.55730/1300-0144.6362

**Published:** 2026-06-01

**Authors:** Burak ÖZBAŞ, Muhammet Mustafa ETLEÇ, Hüseyin DURSUN, Mustafa GÖK, Abdullah Bahadır ÖZ, Alper Celal AKCAN, Ayşa HACIOĞLU, Ökkeş İbrahim KARAHAN, Züleyha Cihan ÖZDAMAR KARACA, Kürşad ÜNLÜHIZARCI

**Affiliations:** 1 Division of Endocrinology and Metabolism, Department of Internal Medicine, Faculty of Medicine, Erciyes University, Kayseri, Turkiye; 2 Department of Radiology, Faculty of Medicine, Erciyes University, Kayseri, Turkiye; 3 Department of General Surgery, Faculty of Medicine, Erciyes University, Kayseri, Turkiye

**Keywords:** Hounsfield unit, computerized tomography, adrenal incidentaloma, adrenal adenoma

## Abstract

**Background/aim:**

Adrenal incidentalomas are increasingly detected during imaging studies performed for various indications, and their frequency in endocrinology practice has risen in recent years. While adenomas account for approximately 80% of adrenal incidentalomas, the remaining 20% include lesions such as primary malignancies, metastases, and pheochromocytomas that should not be overlooked. Given the risks associated with adrenal biopsy, accurate diagnosis with noninvasive methods is essential. In this retrospective study, we evaluated the Hounsfield unit (HU) values and lesion sizes measured on noncontrast computed tomography (CT) to identify a more accurate threshold than the widely used 10 HU.

**Materials and methods:**

In this retrospective study, patients who underwent unilateral adrenalectomy between 2013 and 2022 were evaluated. Noncontrast CT images were assessed for lesion size and attenuation, and statistical analyses included tests for nonnormally distributed data, logistic regression, and ROC analysis to determine the diagnostic performance of mass size and density in differentiating adenoma from nonadenoma lesions.

**Results:**

A total of 106 patients who underwent adrenalectomy for various indications were included, of whom 55 had adenomas and 51 had nonadenoma masses. The mean size of the adenoma versus nonadenoma lesions was 37.2 ± 15 mm and 55.8 ± 30 mm, respectively (p < 0.05), while mean HU values were 11.4 ± 17 and 35.9 ± 14, respectively (p < 0.05). The ROC analysis showed that HU values exhibited strong discriminative ability in distinguishing adenoma from nonadenoma masses (AUC = 0.869). According to Youden’s index, 20 HU was identified as the optimal threshold value. The sensitivity/specificity values for 10 HU, 15 HU, and 20 HU were 98%/56%, 98%/61%, and 92%/82%, respectively.

**Conclusion:**

Within this selected adrenalectomy cohort, adopting a 20 HU threshold instead of the conventional 10 HU value significantly improved specificity in differentiating adenomas from nonadenomas. However, broader prospective validation in unselected incidentaloma populations is warranted before recommending a definitive change in routine clinical guidelines.

## 1. Introduction

Masses detected in the adrenal gland during imaging performed for nonadrenal reasons are called adrenal incidentaloma [1]. The frequency of adrenal incidentalomas in endocrinology practice is gradually increasing due to the more frequent use of imaging methods and the improvements in imaging quality, resulting in a significant workload [2]. The rate of adrenal incidentalomas is 6% of all autopsies and may rise to 10% in the population over the age of 70 [3,4].

Adrenal incidentalomas should be evaluated for both malignancy potential and hormonal functionality from the moment they are diagnosed. It is known that the vast majority of adrenal incidentalomas are benign adrenocortical adenomas and are nonfunctional [5,6]. Although malignant masses seen in the adrenal gland may be of primary adrenal origin, frequently they originate from malignant lesions of other body sites, metastasizing to the adrenal glands. It is important to distinguish whether these adrenal masses are malignant or benign in oncology and endocrine practice. In a patient with known malignant disease, adrenal masses raise the suspicion of metastasis. Adrenal metastases of a malignant tumor result in the patient being considered to have stage IV cancer. However, it is known that the majority of adrenal incidentalomas detected even in patients with known primary malignancy are benign [7]. Although histopathological diagnosis is the gold standard for distinguishing between benign and malignant masses, biopsy is not frequently performed in adrenal masses due to the low accuracy rate and risk of complications; radiological examinations are conducted instead [5,8].

Many different imaging methods are used for the diagnosis of adrenal incidentalomas. These include contrast-enhanced magnetic resonance imaging (MRI), computed tomography (CT) (without contrast, contrast-enhanced, and/or dynamic), and functional imaging performed by nuclear medicine (scintigraphy and positron emission tomography). Among all these, the most widely used imaging technique is noncontrast CT. The attenuation value obtained in noncontrast tomography is expressed as a Hounsfield unit (HU), and its undeniable contribution in distinguishing adenoma and nonadenoma masses has been shown in different publications [5,9,10]. Attenuation value and HU are determined according to the amount of fat contained in the masses, and adrenal masses are interpreted as adenoma or nonadenoma according to the thresholds determined.

There are various publications and guidelines concerning these thresholds. For years, one of the most frequently cited guidelines were those published by the American Association of Clinical Endocrinologists (AACE) and the American Association of Endocrine Surgeons (AAES) [11], in which a HU threshold of ≤10 is recommended to distinguish between adenoma and nonadenoma masses. However, subsequent studies have shown that when the HU threshold is taken as ≤10 to evaluate masses as adenomas, although the mass is actually an adenoma, the HU is calculated higher than 10 due to the low-fat content in the masses, and 10%–40% of adenomas can be misdiagnosed [12,13]. In contrast to the binary approach of the earlier AACE/AAES guidelines, the current 2023 ESE/ENSAT guidelines advocate for a more nuanced risk stratification framework [14]. While an unenhanced CT attenuation of £10 HU remains highly specific for identifying lipid-rich benign adenomas, lesions falling within the 11–20 HU range are now categorized as being in an ‘indeterminate zone’ that necessitates further characterization.

Given this evolving diagnostic landscape, there is a clear need to validate higher threshold values that can optimize clinical decision-making. In the present study, we aimed to determine a more accurate HU threshold to distinguish between adenoma and nonadenoma masses by retrospectively examining all patients who underwent unilateral adrenalectomy in a single tertiary center, utilizing postoperative pathological diagnoses as the gold standard.

## 2. Materials and methods

### 2.1. Patient selection

**Figure 1:**
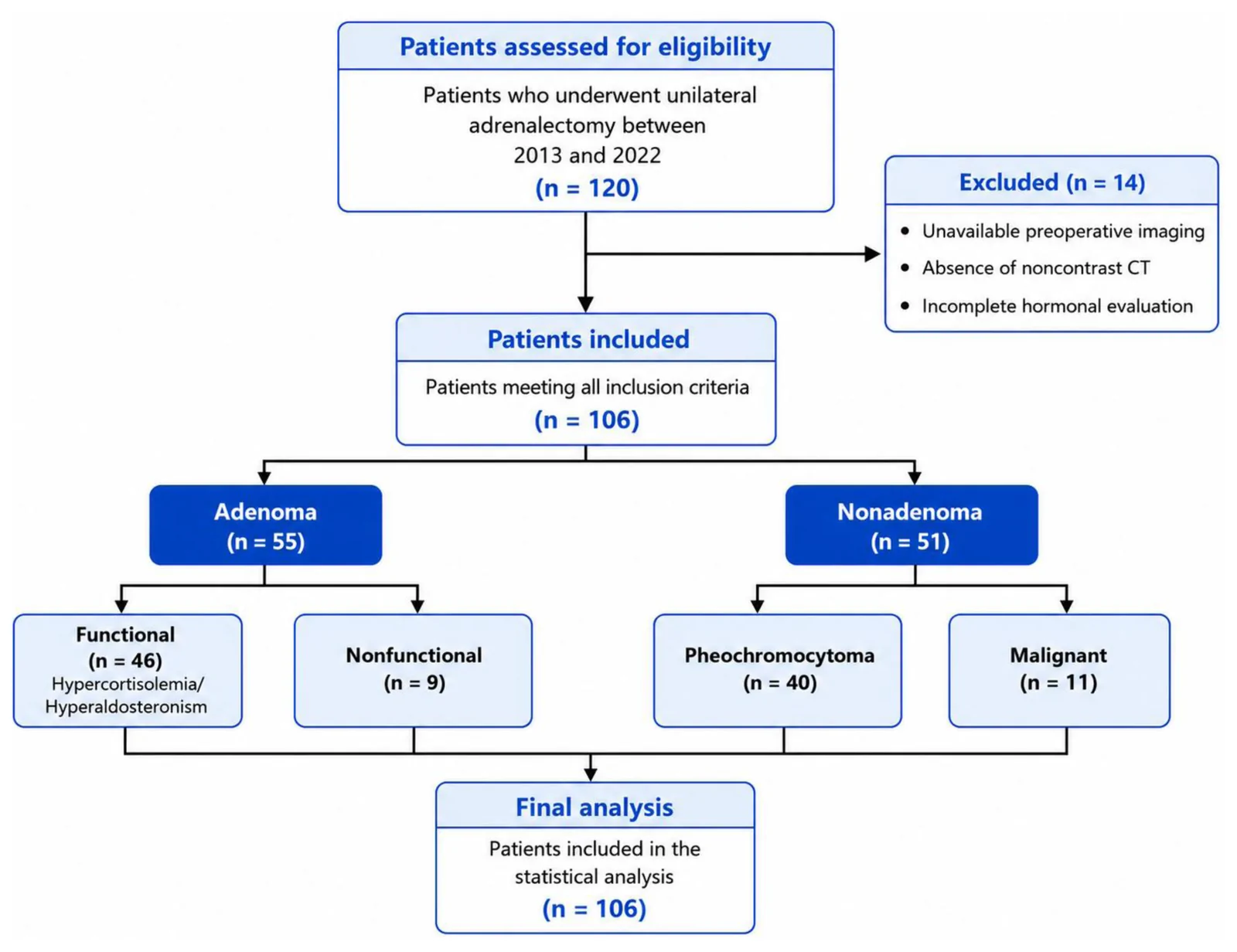
Flow diagram of the study.

We retrospectively evaluated 120 patients who underwent unilateral adrenalectomy for different reasons between 2013 and 2022. All procedures were conducted in accordance with the principles of the Declaration of Helsinki, and our study was approved by the local ethics committee (Erciyes University Faculty of Medicine Clinical Research Ethics Committee) (Decision No. 2023/58, Date 18/01/2023). Patients whose preoperative images were unavailable or did not have a noncontrast CT image and had not undergone hormonal investigations were excluded from the study. As a result, 106 patients were included in the study (Figure 1). To ensure diagnostic clarity, the patients were categorized based on a combination of preoperative biochemical workup and postoperative histopathological diagnosis. Functional adenomas were defined as adrenocortical lesions with histopathological confirmation of adenoma, associated with preoperative biochemical evidence of hormonal hypersecretion. This category included patients with cortisol excess and primary aldosteronism. Cortisol excess was defined as either autonomous cortisol secretion (post-1-mg dexamethasone suppression test [DST] cortisol level >5 μg/dL) or mild autonomous cortisol secretion (post-1-mg DST cortisol between 1.9 and 5 μg/dL), supported by at least one additional biochemical finding (suppressed plasma adrenocorticotropic hormone levels <10 pg/mL, increased 24-h urinary free cortisol, or abnormal late-night salivary cortisol) and/or hypercortisolemia-related comorbidities (resistant hypertension, type 2 diabetes mellitus, and/or osteoporosis). The diagnosis of primary aldosteronism was established in patients with an elevated aldosterone-to-renin ratio and confirmed by a saline infusion test showing a failure of plasma aldosterone suppression (plasma aldosterone concentration > 5 ng/dL).

Pheochromocytomas were identified through both preoperatively elevated biochemical markers (elevated serum and/or 24-h urine metanephrine/normetanephrine) and histopathological confirmation. Malignant masses, including both primary adrenocortical carcinomas and metastases, were diagnosed strictly via histopathological examination. All cases with hormonal excess or suspected malignancy were discussed by a multidisciplinary endocrine board to confirm the indication for adrenalectomy.

### 2.2. Statistical analysis

The data were evaluated using SPSS 23.0. Descriptive statistics are presented as mean ± standard deviation (SD), median (min–max), frequency distribution, and percentage. Although nonparametric tests were applied for inferential analyses, mean ± SD values were also presented to facilitate comparison with previous adrenal imaging literature. The chi-squared test was used for categorical variables. For categorical comparisons involving contingency tables with small expected cell counts, the Fisher–Freeman–Halton exact test was used. Mann–Whitney U and Kruskal–Wallis tests were used since the data for continuous variables did not meet parametric test conditions (normal distribution and homogeneity of variances). Post hoc Bonferroni correction was applied for variables with p < 0.05 in the four-group analysis. Pairwise comparisons were performed using the Mann–Whitney U test. In post hoc analyses, the p-value was adjusted by Bonferroni correction [p < 0.05/6 (0.0083) was considered statistically significant]. To evaluate factors associated with adenoma versus nonadenoma, bivariate logistic regression analyses were initially conducted for each independent variable. Variables with a p-value less than 0.05 in the bivariate analyses were subsequently included in a multivariate logistic regression model. Crude and adjusted odds ratios (cOR and aOR) with 95% confidence intervals were reported. ROC analysis was performed to evaluate the diagnostic performance of HU values and mass size in differentiating adenoma from nonadenoma masses. The optimal threshold was determined according to Youden’s index. In addition, the diagnostic performances of 10 HU and 15 HU were also evaluated for comparison with the conventional threshold recommended in current guidelines and alternative thresholds discussed in previous studies [11,13]. The areas under the curves (AUCs) for density and maximum diameter were calculated and compared using the method described by Hanley and McNeil [15]. The statistical software MedCalc (MedCalc Software Version X, Ostend, Belgium) was used for AUC comparison analyses. A p-value <0.05 was considered statistically significant.

### 2.3. Image analysis

All abdominal CT examinations were performed using a 320-detector CT system (Aquilion ONE, Toshiba Medical Systems). The patients were placed in the supine and feet first position on the couch. No contrast medium was administered. Acquisition parameters were 80 × 0.5 mm detector collimation, 120 kV, 50–550 mA (with automatic modulation), 512 × 512 matrices, 1-mm section thickness, 0.8-mm section interval, and noise index of 10.

The images were reviewed retrospectively by a single radiologist on a picture archiving and communication system (PACS) workstation (Sectra PACS IDS 7, Sweden). Lesion size, laterality (left/right), and attenuation (HU) were recorded. The attenuation of the lesions was measured using a circular or ovoid ROI set that was as large as possible but entirely contained within the boundary of the adrenal lesion to avoid partial volume averaging with the surrounding tissue. Care was taken to avoid areas of visible necrosis, hemorrhage, cystic degeneration, or coarse calcification during ROI placement whenever present. HU measurements were obtained from the axial slice considered most representative of the lesion, using a single ROI measurement for each lesion. In heterogeneous masses, the ROI was preferentially positioned over the solid enhancing-appearing component while avoiding visually nonviable areas. Since image review was performed retrospectively by a single radiologist, interobserver variability and repeat-measurement reproducibility were not assessed.

## 3. Results

Of the 106 patients included, 74 were women and 32 were men. While 46 (43%) patients were operated on due to hormonal hypersecretion, 60 (57%) were operated on due to suspicion of malignancy, pheochromocytoma, or increased mass size. Fifty-five (52%) patients were pathologically diagnosed with adenoma, while 51 (48%) patients were diagnosed with other than adenoma. When patients with a histopathological diagnosis of adrenal adenoma were examined according to their functionality status, 46 (83%) patients were functional and nine (17%) patients had nonfunctional adenoma (Table 1).

When adrenal masses were compared both as adenoma versus nonadenoma masses and in terms of functionality, a significant difference was found between functional adenomas and the other three groups in terms of size (34.4 ± 10 mm vs. 51.2 ± 25 mm, 51.2 ± 26 mm, and 72.4 ± 39 mm, respectively, p < 0.001). A significant difference in density was observed between functional adenomas (9.2 ± 15 HU) and both pheochromocytomas (35.8 ± 15 HU) and malignant masses (36 ± 15 HU) (p < 0.001). When functional and nonfunctional adenomas were compared according to their density in noncontrast CT scans with post-hoc analysis, no statistically significant difference was observed between the two groups regarding the HU value after applying strict Bonferroni correction for multiple pairwise comparisons (9.2 ± 15 vs. 21.7 ± 25, p = 0.039; where the adjusted significance threshold was set at p < 0.0083) (Table 2).

**Figure 2:**
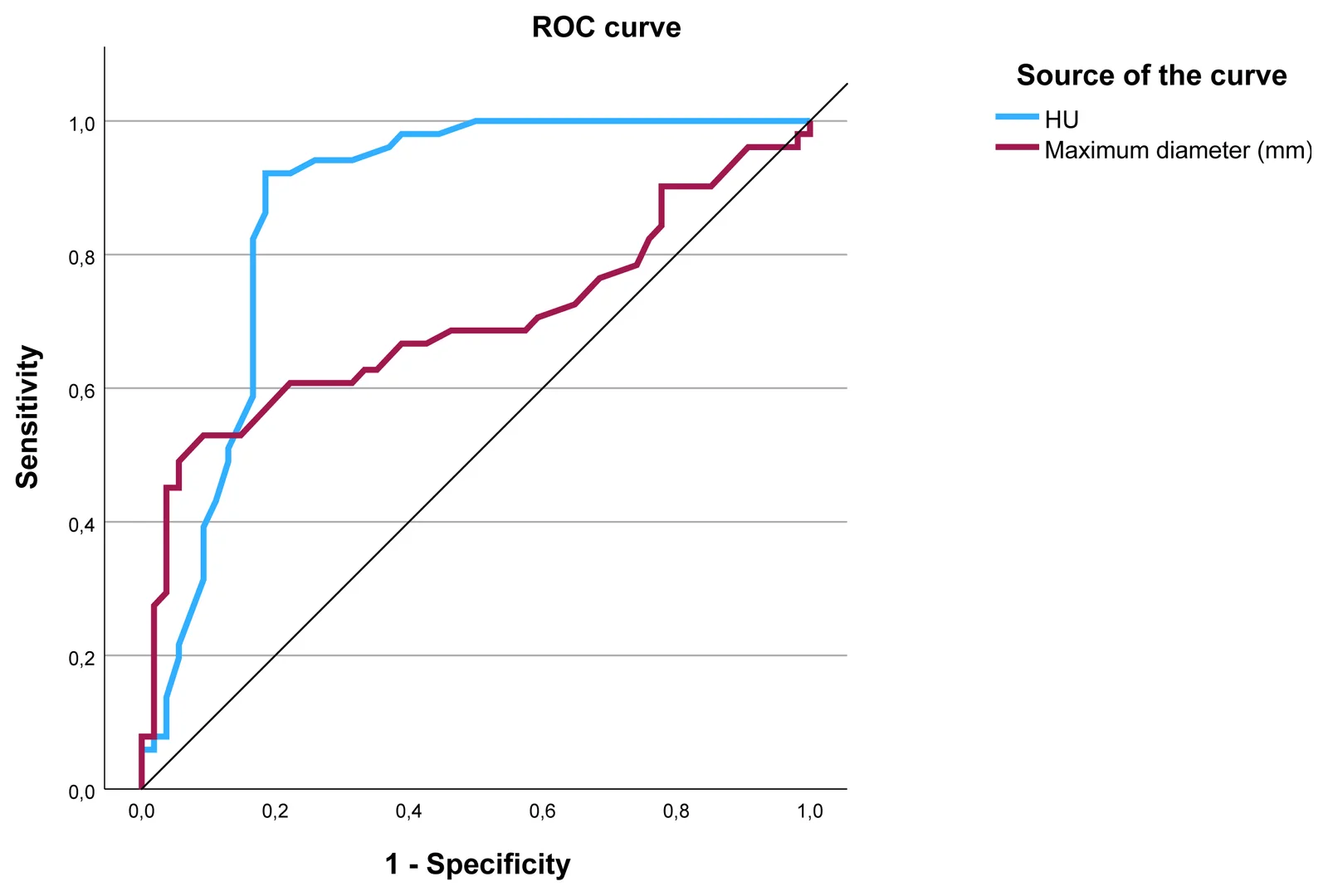
ROC curves demonstrating the diagnostic performance of HU values and maximum diameter in differentiating adenoma from nonadenoma adrenal masses (AUC: 0.869 and 0.699, respectively).

After the evaluation in terms of hormonal functionality, the patients were categorized into two groups according to histopathological diagnosis and analyzed according to HU and size (Table 3). The adenoma group included all adenomas, both functional and nonfunctional, while pheochromocytoma and all malignant masses of the adrenal gland (primary or metastatic) were in the nonadenoma group. The distribution of adenoma, pheochromocytoma, and malignant lesions according to clinically relevant HU categories is presented in Table 4. Among the lesions with HU values ≤10, 96.8% were adenomas. In the clinically indeterminate 11–20 HU interval, 15 lesions were adenomas, one was a pheochromocytoma, and two were malignant. In contrast, the lesions with HU values >20 were predominantly nonadenoma masses, particularly pheochromocytomas and malignant lesions (p < 0.001) (Table 4). The mean mass size was 37.2 ± 15 mm in the adenoma group and 55.8 ± 30 mm in the nonadenoma group. In the ROC analysis performed to find the ideal cut-off point in terms of size in distinguishing between adrenal adenoma and nonadenoma masses, the most appropriate cut-off point was 53.5 mm (AUC 0.699 (0.591–0.807), p < 0.001). When 53.5 mm was taken as the cut-off point, sensitivity and specificity were calculated as 46% and 98%, respectively (Figure 2). When 40 mm was taken as the cut-off point, sensitivity and specificity were calculated as 67% and 61%, respectively.

When comparing the densities on the CT scans of patients diagnosed with adenoma versus those with nonadenoma cases, a significant difference was observed between the two groups (HU value 11.4 ± 17 vs. 35.9 ± 14, p < 0.05). After finding a significant difference between the HU values of the two groups including adenoma and nonadenoma masses, we aimed to determine a new cut-off point that could distinguish between these two groups before surgery. As a result of the ROC analysis, it was determined that the HU value used to determine the density of the masses showed strong discrimination ability in distinguishing between adenomas and nonadenoma masses (AUC = 0.869, p = 0.005). According to Youden’s index, 20 HU was identified as the optimal threshold value balancing sensitivity and specificity. In addition, the diagnostic performances of 10 HU and 15 HU were evaluated for comparison with thresholds commonly used in previous studies and clinical practice [11,13]. When 10 HU was determined as the cut-off point, sensitivity was calculated as 98%, specificity as 56%, positive predictive value as 67%, and negative predictive value as 97%, while the same values for 15 HU were 98%, 61%, 70%, and 97%, respectively, and for 20 HU were 92%, 82%, 83% and 92%, respectively (Table 5).

In the comparison of AUC values (HU values and largest diameter), the difference between the AUCs was 0.194 (95% CI: 0.076–0.311). This difference was significant in the paired ROC curve comparison performed according to the Hanley and McNeil method (z = 3.228, p = 0.0012) (Figure 2).

In addition, bivariate and multivariate logistic regression analysis was performed to evaluate the variables that led to distinction between adenoma and nonadenoma masses. In the bivariate logistic regression analysis, both mass size and density were significantly associated with the diagnosis of adenoma versus nonadenoma (p < 0.001 for both). However, in the multivariate model, only density remained a significant independent factor (aOR = 0.898; 95% CI: 0.858–0.940; p < 0.001), while the largest diameter did not retain significance (p = 0.061). Age was not included in the multivariate model due to its lack of significance in the bivariate analysis (p = 0.707) (Table 6).

## 4. Discussion

Significant differences exist in the distribution of adrenal incidentalomas between clinical and surgical studies. While the rate of adrenal adenoma diagnosis reaches 80% in clinical studies, it is around 50% in surgical series [16]. The reason for this difference is that the vast majority of adrenal incidentalomas are nonfunctioning and small, thus lacking indications for surgery and requiring only routine follow up. In the cases included in our study, the histopathological diagnosis of adrenal adenoma was detected at a rate similar to that reported in other surgical series (51.8%).

We also think that the difference in size between the groups is related to the course of the diseases. While adrenal hypercortisolemia/hyperaldosteronism cases are usually operated on early due to clinical findings, nonfunctioning adenomas can remain silent for a very long time and reach large sizes without being noticed, while primary or secondary malignancies can reach very large sizes in a very short time.

The main guidelines published on adrenal incidentaloma in the last decade focus on four questions: 1) Assessment of the risk of malignancy, 2) Identification and management of MACS, 3) need for surgical treatment and need for surgery when necessary, 4) follow-up method of patients who did not receive surgical treatment [14].

Our primary aim in the present study was to find a practical answer to the first question. In our analysis, as expected, a significant difference was found between adenoma and nonadenoma masses in terms of HU value (11.3 ± 18 vs. 35.9 ± 15, p < 0.05). We evaluated three clinically relevant cut-off points (10 HU, 15 HU, and 20 HU) to compare their diagnostic performances in distinguishing adenoma from nonadenoma masses. Sensitivity was 98% for both the 10 HU and 15 HU thresholds, whereas specificity was highest at the 20 HU threshold (specificity 56%, 61%, and 82%, respectively).

An increase in the size of adrenal masses is known to be associated with an increased risk of malignancy. CT is the most widely used imaging modality and can detect adrenal masses as small as 5 mm. Similar to the current literature, in our study the ideal threshold determined for distinguishing malignant from benign masses was 40 mm [17]. However, as expected, high sensitivity and specificity percentages were not achieved (67% and 61%, respectively). These rates are also similar to those in other publications in the literature [13]. Although size is important in distinguishing possible malignant from benign adrenal masses, it is often insufficient alone. Distinguishing between malignant and benign adrenal masses, not only the size but also the imaging features of the mass on CT (density, shape, and margins) may also contribute [18]. Contrast-enhanced examinations, in particular, may provide an additional contribution.

Tumor size is an important parameter in assessing the risk of malignancy in many tumors. However, analysis of our data showed that adrenal mass size alone was limited in predicting the risk of malignancy in the multivariate model. This suggests that HU measurement should maintain its priority in distinguishing adenomas from nonadenomas in adrenal masses. However, this recommendation should not mean that tumor size should be neglected in the evaluation. Instead, we think that including it alongside HU measurement will provide additional support. For instance, an adrenal lesion >40 mm with HU >20 is far more suspicious for nonadenoma etiology than a lesion that only meets one of these criteria. Therefore, rather than relying on size or density in isolation, integrating both features into a risk stratification algorithm may enhance diagnostic accuracy and help guide decisions regarding follow-up versus surgical intervention.

The limitations of our study include its retrospective design and the lack of venous phase postcontrast enhanced CT examination in the patients. Although the HU value obtained from noncontrast CT scans alone provides a high sensitivity/specificity ratio, Kirsch reported that adding the absolute percent contrast washout measurement greater than 60% to the HU value, which is considered in favor of adenoma, increases these percentages even more [13].

A major limitation of our study is its retrospective design, with attenuation measurements performed by a single radiologist without repeat measurements or formal assessment of interobserver reproducibility. Particularly in heterogeneous adrenal lesions, the strategy and precise location of ROI placement may materially influence the measured HU values, representing a potential source of variability. Furthermore, because our cohort was entirely surgery-based, it predominantly consisted of functional adenomas rather than the more commonly encountered nonfunctioning incidentalomas monitored in routine clinical practice, which may limit the generalizability of our findings to the broader population of incidentally discovered adrenal masses.

In our cohort, a threshold of 20 HU demonstrated higher specificity than the conventional 10 HU threshold recommended by current guidelines. Using 20 HU as the threshold may increase specificity and potentially reduce the risk of unnecessary surgery for adrenal incidentalomas. However, this carries the risk of missing the diagnosis of adrenal adenomas with low fat content. This necessitates the inclusion of the patient’s clinical context, tumor size, and, if necessary, advanced imaging techniques in the diagnostic process, in addition to HU measurement.

In conclusion, our study indicates that the HU measurement, whose diagnostic value has been known for many years in distinguishing adenoma from nonadenoma masses, may be more useful if the threshold value is set at 20 instead of 10. Although the fact that all our patients had histopathological diagnoses is a strength of the present study, it should not be forgotten that only a small portion of the large number of patients presenting with adrenal incidentalomas underwent surgery. The majority of patients with extraadrenal malignancies do not undergo metastasis surgery, and many benign-appearing masses that do not meet the criteria for surgical necessity and are likely to be adenomas are monitored only with annual hormone monitoring once imaging techniques show that they are not growing. Prospective studies that use both noncontrast and contrast-enhanced imaging are needed to obtain more definitive conclusions.

## Figures and Tables

**Table 1:** Sociodemographic characteristics of the patients.

Variable	n (%)

**Sex**	
Female	74 (70%)
Male	32 (30%)

**Surgery reason**	
Functionality (hormonal hypersecretion)	86 (81%)
Functional adenomas	46 (43%)
Pheochromocytoma	40 (38%)
Suspicion of primary adrenal malignancy/metastasis or large mass size	20 (19%)

**Diagnosis**	
Adenoma	55 (52%) (46 functional/9 nonfunctional)
Nonadenoma	51 (48%)

**Table 2:** Characteristics of masses in patients undergoing adrenalectomy, by functionality and type.

	Hypercortisolemia/hyperaldosteronism (n=46)	Pheochromocytoma (n=40)	Nonfunctional adenoma (n=9)	Malignant lesions (n=11)	p

**Sex**, n (%)					
Female	37 (50%)	23 (31.1%)	8 (10.8%)	6 (8.1%)	0.044
Male	9 (28.1%)	17 (53.1%)	1 (3.1%)	5 (15.6%)	

**Age**					
mean±SD	52.6±11	51.4±12	56±11	56.8±6	0.390
median (min–max)	54(22–75)	51 (26–74)	59 (34–72)	58 (48–65)	

**BMI**,					
mean±SD	32.4±7	26.2±3	27.8±3	36.7±28	<0.001^a^
median (min–max)	31 (23–48)	25 (22–37)	27 (25–35)	28 (20–120)	

**Maximum diameter (mm)**					
mean±SD	34.4±10	51.2±25	51.2±26	72.4±39	<0.001^b^
median (min–max)	35 (9–55)	47 (11–156)	48 (12–110)	79 (8–120)	

**Density (HU)**					
mean±SD	9.2±15	35.8±15	21.7±25	36±15	<0.001^c^
median (min–max)	6 (−18–45)	33 (10–85)	17 (16–65)	37 (17–73)	

HU: Hounsfield unit, BMI: body mass index

a Difference in BMI was detected between pheochromocytoma and functional masses (p < 0.001).

b Significant difference in size between functional adenomas and the other three groups (nonfunctioning adenoma, pheochromocytoma, and malignant masses) (p < 0.001, p = 0.008, p = 0.008).

c Significant differences in density were detected between functional adenomas and nonadenoma masses (pheochromocytoma and malignant masses) (p < 0.001).

**Table 3:** Characteristics of patients who underwent surgery with the diagnosis of adenoma or nonadenoma mass.

Variable	Adenoma	Nonadenoma

**Sex, n (%)**		
Female	45 (60.8)	29 (39.2)
Male	10 (31.3)	22 (68.8)

**Age**		
mean±SD	53.2±11.5	52.3±11.7
median (min–max)	55 (22–75)	52 (26–74)

**Maximum diameter (mm)**		
mean±SD	37.2±15.3	55.8±30.1
median (min–max)	36 (9–110)	53 (8–156)

**Density (HU)**		
mean±SD	11.4±17.8	35.9±14.8
median (min–max)	9 (–18 to 65)	34 (10–85)

HU: Hounsfield unit

**Table 4:** Distribution of adenoma, pheochromocytoma, and malignant lesions according to clinically relevant HU categories.

	Adenoma (n=55)	Pheochromocytoma (n=40)	Malignant (n=11)	p
HU ≤10	30 (96.8%)	1 (3.2%)	0 (0.0%)	<0.001
HU 11–20	15 (83.3%)	1 (5.6%)	2 (11.1%)	
HU >20	10 (17.5%)	38 (66.7%)	9 (15.8%)	

HU: Hounsfield unit

**Table 5:** Diagnostic efficiencies of HU values obtained with noncontrast CT imaging at different threshold points for the distinction of adenomas and nonadenoma masses.

	Sensitivity	Specificity	PPV	NPV
HU >10	98%	56%	67%	97%
HU >15	98%	61%	70%	97%
HU >20	92%	82%	83%	92%

HU: Hounsfield unit, PPV: positive predictive value, NPV: negative predictive value

**Table 6:** Bivariate and multivariate logistic regression analysis of age, mass size, and density in differentiating adenoma from nonadenoma masses.

	Bivariate model cOR (95%CI)	p	Multivariate model aOR (95%CI)	p
Age	1.007 (0.971–1.044)	0.707	–	-
Maximum diameter (mm)	0.953 (0.928–0.980)	<0.001	0.970 (0.939–1.001)	0.061
Density	0.891 (0.852–0.933)	<0.001	0.898 (0.858–0.940)	<0.001

The dependent variable was coded as 1 for adenoma and 0 for nonadenoma masses
